# Thwarting endogenous stress: BRCA protects against aldehyde toxicity

**DOI:** 10.15252/emmm.201708194

**Published:** 2017-08-23

**Authors:** Arnab Ray Chaudhuri, André Nussenzweig

**Affiliations:** ^1^ Department of Molecular Genetics Erasmus University Medical Center Rotterdam The Netherlands; ^2^ Laboratory of Genome Integrity National Cancer Institute National Institutes of Health Rockville MD USA

**Keywords:** Cancer

## Abstract

Homologous recombination (HR) and the Fanconi Anemia (FA) pathways constitute essential repair pathways for DNA damage, which includes DNA double‐stranded breaks (DSB) and inter‐strand cross‐links (ICL), respectively. Germline mutations affecting a single copy of the HR factors BRCA1 and BRCA2 predispose individuals to cancers of the breast, ovary, prostate, and pancreas. Cells deficient for BRCA proteins display high levels of genome instability due to defective repair of endogenous DSBs and are also exquisitely sensitive to DNA‐damaging agents. In addition to their roles in repair of DSBs and ICLs, HR and FA proteins have a genetically separable function in the protection of stalled DNA replication forks from nuclease‐mediated degradation (Schlacher *et al*, 2012). Although it has been hypothesized that loss of functional HR and ICL repair is the primary cause of cancer in BRCA‐ and FA‐deficient patients (Prakash *et al*, 2015), the contribution of replication fork instability associated with the degradation of nascent DNA remains unclear. Two recent papers explain how endogenous toxins render cells vulnerable to genomic instability, which explains how the BRCA/FA pathway suppresses tumorigenesis (Tacconi *et al*, 2017; Tan *et al*, 2017).

Reactive aldehydes are intermediates or final products of endogenous metabolic processes, which if not catabolized, may form DNA‐protein cross‐links (DPC) and ICLs. Seminal work from the laboratory of K.J. Patel has shown that FA pathway is essential to counteract these adducts, which may form ICLs (Langevin *et al*, 2011). Combined loss of FA pathway protein FANCD2 and aldehyde catabolism enzymes leads to an increased DNA damage load, resulting in bone marrow failure and malignancies in mutant mice (Langevin *et al*, 2011). Mutations in ALDH2, the enzyme required for acetaldehyde metabolism, are also associated with predisposition to cancer signifying its critical role in the removal of genotoxic metabolic intermediates (Cai *et al*, 2015). However, the mechanisms by which aldehyde toxicity acts as a driver of tumorigenesis and whether BRCA1 and BRCA2 protect against these lesions remained unclear.

New work from Madalena Tarsounas shows that inactivation of HR factors BRCA1, BRCA2, and RAD51 in cell lines results in hypersensitization to either genetic ablation or chemical inhibition of the acetaldehyde metabolizing enzyme ALDH2 (Tacconi *et al*, 2017). Moreover, treatment with acetaldehyde in BRCA1‐ and BRCA2‐deficient mouse tumor models and patient‐derived tumor xenografts *in vivo* results in regression of tumors. How does a DNA‐damaging agent like acetaldehyde lead to the inhibition of tumor growth? Assuming that acetaldehyde induces cross‐links, DSB intermediates might be expected to form after the replication fork collides with the lesion, which would require error‐free repair by HR.

A well‐known mechanism of chemotherapy resistance in BRCA‐deficient tumors occurs through the restoration of HR (Lord & Ashworth, 2013). One would therefore predict that restoration of HR functions would result in resistance to acetaldehyde. However, Tacconi *et al* (2017) find that BRCA1/53BP1‐deficient tumors, which are HR proficient, are exquisitely sensitive to acetaldehyde treatments *in vivo*. This surprising observation might be explained by three non‐mutually exclusive mechanisms (Fig 1): (i) While HR functions are restored, BRCA1 and 53BP1 double‐deficient cells are not protected from replication fork degradation (Ray Chaudhuri *et al*, 2016), and acetaldehyde treatment induces replication stress in these cells (Tacconi *et al*, 2017); (ii) BRCA1 has a role in cross‐link repair upstream of its role in HR, and BRCA1/53BP1‐deficient cells remain hypersensitive to DNA cross‐links (Bunting *et al*, 2012); (iii) a recent report from the laboratory of Ashok Venkitaraman has shown that exposure to aldehydes causes selective degradation of BRCA2 resulting in “induced haploinsufficiency” for BRCA2 (see below) (Tan *et al*, 2017). The reduction in the levels of BRCA2 upon acetaldehyde treatments could result in HR defects that re‐sensitize BRCA1/53BP1‐deficient tumors, which were initially proficient for HR. In summary, these studies suggest that BRCA‐deficient tumors could be sensitized by disrupting the aldehyde metabolism pathway. Furthermore, aldehyde might be effective in BRCA‐deficient tumors, which have re‐established HR and become resistant to chemotherapeutic agents such as PARP inhibitors.

**Figure 1:**
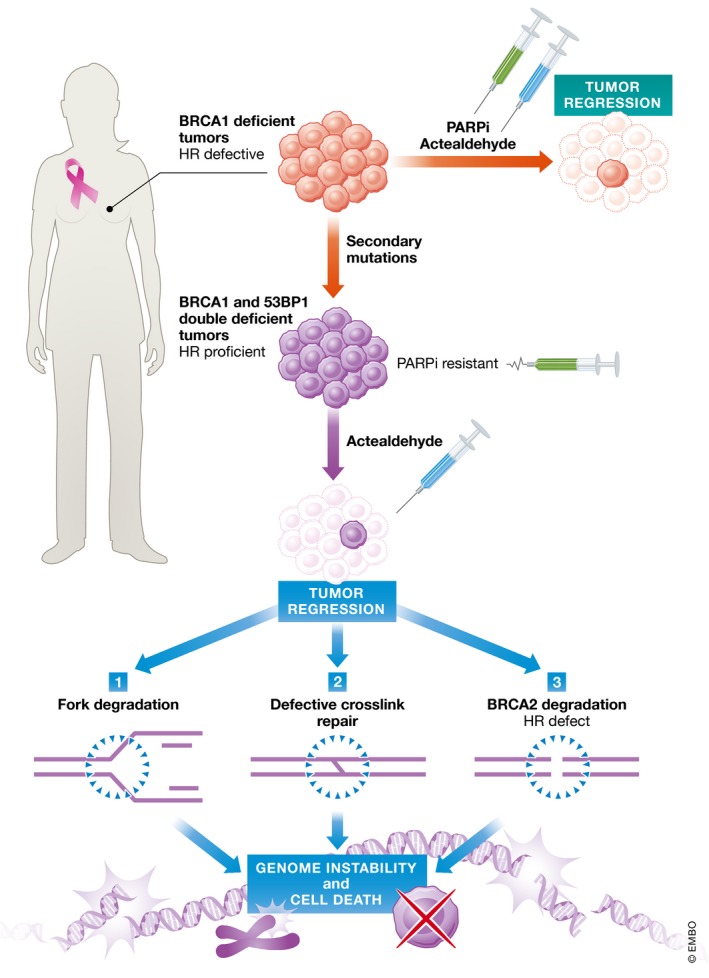
Model for aldehyde sensitivity in BRCA‐deficient tumors Breast tumors with BRCA1 mutations are sensitive to DNA‐damaging agents like PARP inhibitors (PARPi) and acetaldehyde due to defective homologous recombination (HR). However, these tumors have a high propensity to develop resistance to drugs by acquiring secondary mutations in BRCA1 or in factors like 53BP1 and that restore HR. Tan *et al* (2017) show that these PARPi‐resistant, HR‐proficient tumors are sensitive to acetaldehyde treatments. The molecular mechanisms underlying this sensitivity can be explained by three mutually non‐exclusive mechanisms: (1) BRCA1 and 53BP1 double mutants are still defective for replication fork protection although they are HR proficient. This could result in genome instability and loss of cellular viability. (2) BRCA1‐ and 53BP‐deficient cells are defective for cross‐link repair as BRCA1 has roles in the repair of cross‐links upstream of HR. (3) Acetaldehyde treatments could result in selective degradation of BRCA2 proteins in these HR‐proficient cells rendering them HR deficient and thus making them hypersensitive to acetaldehyde treatments. These mechanisms in concert could account for increased genome instability and cell death observed in the HR‐proficient PARPi‐resistant tumors.

However, treatment with drugs such as acetaldehyde can have both favorable as well as adverse consequences. A recent report from Venkitaraman and colleagues suggests that exposure to endogenous aldehydes could also drive tumorigenesis in BRCA2 heterozygous carriers (Tan *et al*, 2017). They show that formaldehyde exposure of cells harboring heterozygous pathogenic mutations in BRCA2 leads to increased genome instability. This genome instability arises from replicative stress induced by formaldehyde, which results in degradation of nascent DNA. The replication fork instability in BRCA2 heterozygous cells is caused by the selective proteasomal degradation of BRCA2 protein upon formaldehyde exposure. Interestingly, the increased genome instability in BRCA2 heterozygous cells induced by exposure to formaldehyde does not result in loss of viability. This could be because BRCA2 haploinsufficient cells can still partially repair DSBs through HR. Tolerable but persistent levels of DNA damage could thereby be an initiating event in tumorigenesis.

In conclusion, therefore, these reports have shed light on two important questions in the DNA repair field: (i) What is the contribution of HR and replication fork stability in preventing tumorigenesis and maintaining cellular viability, and (ii) how do individuals with BRCA heterozygosity develop tumors? HR activity is essential for the survival of organisms. For example, mice deficient for BRCA1 and BRCA2 display embryonic lethality. Embryonic lethality in BRCA1‐deficient mice can be rescued by the deficiency of 53BP1, which results in restoration of HR, whereas replication fork protection contributes to genome stability but does not lead to organismal viability (Ray Chaudhuri *et al*, 2016). The role of HR in preventing tumorigenesis is less clear. Cells from BRCA heterozygous carriers support normal HR activity, yet are haploinsufficient for protecting the genome from unstable replication forks (Pathania *et al*, 2014). Replication stress but not HR deficiency is also characteristic of BRCA2 mutations in mice which disrupt interactions with PALB2, a protein which controls BRCA2 functions in HR. Mono‐allelic mutations in *PALB2* increase the risk of cancer, and biallelic mutations cause FA (Hartford *et al*, 2016). The mutant mice with disrupted BRCA2/PALB2 interaction are viable but exhibit high rates of tumorigenesis (Hartford *et al*, 2016). In conclusion, persistent replication stress due to endogenous or environmental toxins such as reactive aldehydes may be tumorigenic in cells partially compromised for BRCA function. Since a high percentage of the East Asian population carries a dominant‐negative allele of the aldehyde‐catalyzing enzyme ALDH2, studies investigating cancer risk associated with BRCA1 and BRCA2 mutations in this population will be important.
